# Complete Genome Sequences of Two Type 3 Porcine Circoviruses, WB17KW and WB20GG, Isolated from Korean Wild Boar

**DOI:** 10.1128/MRA.01386-20

**Published:** 2021-02-11

**Authors:** Sok Song, Kyu-Nam Park, SeEun Choe, Ra Mi Cha, Jihye Shin, Bang-Hun Hyun, Dong-Jun An

**Affiliations:** a Virus Disease Division, Animal and Plant Quarantine Agency, Gimchen, Gyeongbuk-do, Republic of Korea; KU Leuven

## Abstract

Porcine circovirus type 3 (PCV3) in domestic pigs was first reported in South Korea in 2017. Here, we report the first complete genome sequences of two PCV3 strains isolated from Korean wild boar, which enhance our understanding about the genetic relatedness of PCV3 in domestic pigs and wild boar.

## ANNOUNCEMENT

**FIG 1:**
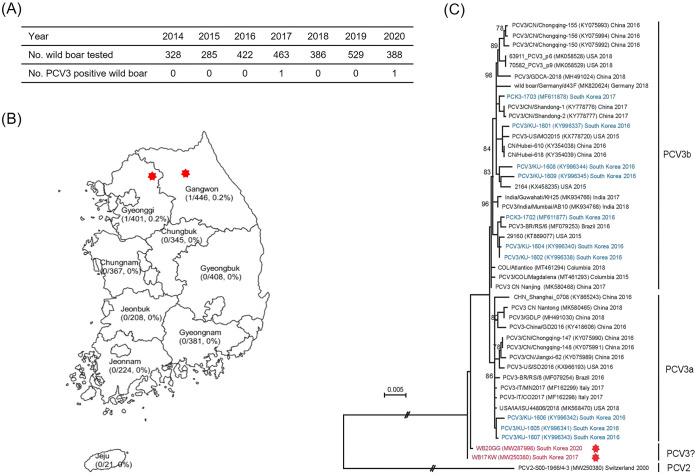
Occurrence by year and region and phylogenetic analysis of PCV3-positive Korean wild boar. Detection of PCV3 for 7 years (2014 to 2020) (A) and by region (B). (C) Phylogenetic tree based on the complete genomes of two Korean wild boar PCV3 strains and 42 PCV3 reference strains. The tree was constructed using the maximum-likelihood method (based on the Tamura-Nei model) with bootstrap values calculated from 1,000 replicates. The scale bar indicates nucleotide substitutions per site. Two Korean wild boar PCV3 strains (WB17KW and WB20GG) are marked with red stars, and Korean domestic pig PCV3 strains are indicated with blue type.

Porcine circovirus type 3 (PCV3), a single-stranded and small circular DNA virus, belongs to the genus *Circovirus* and the family *Circoviridae*. PCV3 appeared suddenly in many countries (United States, China, United Kingdom, Poland, Brazil, and South Korea) between 2016 and 2017 (1). Some pigs infected with PCV3 were asymptomatic, but most of them showed various symptoms, such as reproductive disorders, dermatitis, nephropathy syndrome, diarrhea, congenital tremor, and respiratory disorders (1–3). For this study, we used 2,081 out of 12,318 blood samples of wild boar which were previously collected from all over South Korea between 2014 to 2020 during a surveillance campaign for classical swine fever virus. Total DNA was extracted, and PCV3 positivity was determined by conventional PCR (4). Specifically, total DNA was extracted using the DNeasy blood and tissue kit (Qiagen, USA) and amplified with four specific primer pairs (4) using the Profi *Taq* PCR PreMix kit (bioneer, South Korea), and the PCR products were sequenced by using the Sanger sequencing method. The generated sequencing data were assembled using the CLC Main Workbench software and were aligned using the ClustalX2 program. Open reading frames and sequence identity were analyzed by the BioEdit version 7.2.5 program. A phylogenetic tree was constructed using the maximum-likelihood (ML) method and bootstrap analysis (*n* = 1,000) via the MEGA 6.0 software. As a result, it was confirmed that two samples collected in Gangwon, South Korea, in 2017 and in Gyeonggi, South Korea, in 2020 were PCV3 positive (Fig. 1A and B). These two strains were designated WB17KW and WB20GG, the size of each nucleotide sequence was 2,000 bp, and the similarity between the two strains was 99.75%. The A/T and G/C ratios of these two strains are 51% and 49%, respectively. Two major open reading frames (ORF1 and ORF2) could be identified in both Korean wild boar PCV3 strains. In an ML tree, Korean domestic pig PCV3 strains belonged to both PCV3a and PCV3b clusters, but the two Korean wild boar PCV3 strains clustered separately from the PCV3a or PCV3b clusters (Fig. 1C). In Brazil, wild boars are susceptible to PCV3 infection, and a high detection ratio (42.6%) was reported in wild boar serum samples from 2004 to 2018 (5). This finding suggests that PCV3, which infects Brazilian wild boar, can spread to other wild boar and lead to long-term infection (5). In Italy, PCV3 from Sardinian suids was detected at a rate of 77.4% in free-ranging pigs and 61.5% in wild boar (6). This finding indicates that free-ranging pigs can act as an important reservoir for transmission to wild boar. In this study, we first report two PCV3 strains (WB17KW and WB20GG) from South Korean wild boars, but they are most likely not derived from domestic pigs due to genetic differences with PCV3 strains from domestic pigs. Therefore, it is presumed that these PCV3 strains either migrated from North Korea to South Korea or that they have been circulating in the wild boar population for a long time, without being detected. Further studies are needed for the characterization of these PCV3 strains.

### Data availability.

The complete genome sequences of WB17KW and WB20GG were deposited in GenBank under accession numbers MW250380 and MW287998.
